# Cross-Cultural Validation of a New Version in Spanish of Four Items of the *Preventive COVID-19 Infection Behaviors Scale* (PCIBS) in Twelve Latin American Countries

**DOI:** 10.3389/fpsyg.2021.763993

**Published:** 2021-11-16

**Authors:** Tomás Caycho-Rodríguez, Lindsey W. Vilca, Pablo D. Valencia, Carlos Carbajal-León, Andrea Vivanco-Vidal, Daniela Saroli-Araníbar, Mario Reyes-Bossio, Michel White, Claudio Rojas-Jara, Roberto Polanco-Carrasco, Miguel Gallegos, Mauricio Cervigni, Pablo Martino, Diego Alejandro Palacios, Rodrigo Moreta-Herrera, Antonio Samaniego-Pinho, Marlon Elías Lobos-Rivera, Ilka Franco Ferrari, Carmen Flores-Mendoza, Andrés Buschiazzo Figares, Diana Ximena Puerta-Cortés, Ibraín Enrique Corrales-Reyes, Raymundo Calderón, Bismarck Pinto Tapia, Walter L. Arias Gallegos

**Affiliations:** ^1^Facultad de Ciencias de la Salud, Universidad Privada del Norte, Lima, Peru; ^2^Departamento de Psicología, Universidad Peruana Unión, Lima, Peru; ^3^Facultad de Estudios Superiores Iztacala, Universidad Nacional Autónoma de México, Tlalnepantla de Baz, Mexico; ^4^Facultad de Psicología, Universidad Peruana de Ciencias Aplicadas, Lima, Peru; ^5^Facultad de Ciencias Humanas y Educación, Universidad Peruana Unión, Lima, Peru; ^6^Departamento de Psicología, Facultad de Ciencias de la Salud, Universidad Católica del Maule, Talca, Chile; ^7^Cuadernos de Neuropsicología, Rancagua, Chile; ^8^Pontificia Universidade Católica de Minas Gerais, Belo Horizonte, Brazil; ^9^Consejo Nacional de Investigaciones Científicas y Técnicas, Buenos Aires, Argentina; ^10^Centro Interdisciplinario de Investigaciones en Ciencias de la Salud y del Comportamiento, Universidad Adventista del Plata, Consejo Nacional de Investigaciones Científicas y Técnicas, Rosario, Argentina; ^11^Centro de Investigación en Neurociencias de Rosario, Facultad de Psicología, Universidad Nacional de Rosario, Rosario, Argentina; ^12^Centro de Desarrollo Humano, Universidad Mariano Gálvez de Guatemala, Guatemala, Guatemala; ^13^Escuela de Psicología, Pontificia Universidad Católica del Ecuador, Ambato, Ecuador; ^14^Carrera de Psicología, Facultad de Filosofía, Universidad Nacional de Asunción, Asunción, Paraguay; ^15^Escuela de Psicología, Facultad de Ciencias Sociales, Universidad Tecnológica de El Salvador, San Salvador, El Salvador; ^16^Laboratory of Individual Differences Assessment, Post-Graduation Program in Neuroscience, Universidade Federal de Minas Gerais, Belo Horizonte, Brazil; ^17^Centro de Estudios Adlerianos, Instituto Alfred Adler Uruguay, Montevideo, Uruguay; ^18^Programa de Psicología, Universidad de Ibagué, Ibagué, Colombia; ^19^Servicio de Cirugía Maxilofacial, Hospital General Universitario Carlos Manuel de Céspedes, Universidad de Ciencias Médicas de Granma, Bayamo, Cuba; ^20^Carrera de Psicología, Facultad de Ciencias de la Salud, Universidad del Valle de México, Ciudad de México, Mexico; ^21^Carrera de Psicología, Universidad Católica Boliviana San Pablo, La Paz, Bolivia; ^22^Departamento de Psicología, Universidad Católica San Pablo, Arequipa, Peru

**Keywords:** COVID-19, preventive behaviors, invariance, Latin America, countries

## Abstract

The invariance of the Preventive COVID-19 Infection Behaviors Scale (PCIBS) was evaluated in 12 Latin American countries (Argentina, Bolivia, Chile, Colombia, Cuba, Ecuador, El Salvador, Guatemala, Mexico, Paraguay, Peru, and Uruguay). A total of 5183 people from the aforementioned countries participated, selected using the snowball sampling method. Measurement invariance was assessed by multigroup confirmatory factor analysis (MG-CFA) and Multi-Group Factor Analysis Alignment (CFA-MIAL). In addition, item characteristics were assessed based on Item Response Theory. The results indicate that the original five-item version of the PCIBS is not adequate; whereas a four-item version of the PCIBS (PCIBS-4) showed a good fit in all countries. Thus, using the MG-CFA method, the PCIBS-4 achieved metric invariance, while the CFA-MIAL method indicated that the PCIBS-4 shows metric and scalar invariance. Likewise, the four items present increasing difficulties and high values in the discrimination parameters. The comparison of means of the PCIBS-4 reported irrelevant differences between countries; however, Mexico and Peru presented the highest frequency of preventive behaviors related to COVID-19. It is concluded that the PCIBS-4 is a unidimensional self-report measure which is reliable and invariant across the twelve participating Latin American countries. It is expected that the findings will be of interest to social and health scientists, as well as those professionals directly involved in public health decision making.

## Introduction

At the time of writing, more than 39,604,000 cases of COVID-19 have been reported in Latin America and the Caribbean and more than 1,331,000 have died. Although most Latin American countries have initiated the COVID-19 vaccination process, the spread of COVID-19 in this region has made people’s health behaviors, especially preventive behaviors, important to reduce the rate of disease transmission (Chang et al., 2020a). Health-promoting behaviors are those aimed at maintaining or improving health, which are particularly important in limiting the spread of communicable diseases (Toussaint et al., 2020). In this type of diseases, viruses or bacteria are transmitted from one person to another through different means, such as air, surfaces or body fluids (Weston et al., 2018).

Regarding COVID-19, there are a set of suggested behaviors to prevent the spread of the disease worldwide, such as: washing hands, avoiding touching the face with unwashed hands and close contact with sick people, staying home if the person has symptoms of the disease, covering the mouth and nose with a tissue or arm when coughing or sneezing, washing hands or wiping them with hand sanitizer after coughing or sneezing, wearing masks and face shields, and cleaning and disinfecting frequently touched surfaces (Toussaint et al., 2020). However, while preventive behaviors effectively delay transmission of the virus, their excessive practice can also lead to sedentary behaviors, lack of physical activity, and a host of psychological problems such as unnecessary anxiety (Ye et al., 2020), which could, in turn, cause an increase in alcohol and other drug use (Shatri et al., 2020). Likewise, it is likely that people with higher levels of anxiety may engage in behaviors such as excessive avoidance or persistent, repetitive and unnecessary seeking of medical reassurance; whereas, people with low levels of anxiety tend not to engage in basic hygiene behaviors or other recommended health behaviors, as they do not perceive any notable risk to their health (Velikonja et al., 2020). Governments have also differed in the way they have introduced actions to control behavior; in the severity of the limitations they have imposed; in the amount of time they were enforced; and in government policies to help people make the necessary changes. Also, countries with strict social norms and penalties for those who break them, such as China and Japan, have imposed more preventive health behaviors, at the individual and community levels, as opposed to countries with more permissive social norms, such as Italy and Brazil (Van Bavel et al., 2020). Despite variations between countries, there are also commonalities, such as the use of masks, hand washing and social distancing (Breakwell et al., 2021).

This scenario poses a significant challenge for scientists and practitioners in understanding how to ensure cooperation and compliance in individual countries (Clark et al., 2020). Generating an effective pandemic response requires clear and reliable monitoring. Identifying those who comply with recommended preventive behaviors and the variables that predict compliance are crucial to guide educational practices and identify those sectors of the population at greatest risk for the spread of the disease (Toussaint et al., 2020; Plohl and Musil, 2021). Therefore, it is important to have instruments that provide valid and reliable information on the health behaviors needed to prevent and limit the spread of communicable diseases such as COVID-19.

Adequate measurement of COVID-19 preventive behaviors is not easy, especially as these behaviors may vary over time, between different age groups, degree of vulnerability to COVID-19 or changes in public health guidance and lifting of previous restrictions in different countries, which would mean that prevention behaviors need to adapt to the new social and political context (Daoust, 2020; Breakwell et al., 2021). Likewise, previous studies that have examined preventive behaviors against COVID-19 have used a variety of measures, some derived directly from government guidelines (Toussaint et al., 2020; Vally, 2020), others adapted from instruments used in previous epidemics, such as SARS (Yıldırım and Güler, 2020) or some focused on assessing only one or two preventive behaviors, such as the use of face masks or hand washing. On the other hand, the questions also vary, with some referring to intentions, others to actual behaviors, past and current behaviors, as well as others focused on the likelihood of adopting the behavior, and some even requiring a definite answer, such as yes or no (Breakwell et al., 2021). In this sense, the different way of measuring preventive behaviors makes it difficult to compare results across studies.

Therefore, it is important to have a measure of COVID-19 preventive behaviors that can be used during the pandemic in different cultural contexts, such as Latin America. One of the instruments that can fulfill this role is the Preventive COVID-19 Infection Behaviors Scale (PCIBS; Chang et al., 2020b), because it was developed based on the preventive behaviors recommended by the World Health Organization [WHO] (2020) to avoid COVID-19 infection. The PCIBS was developed in Taiwan and is comprised of five items, which cluster satisfactorily on a single dimension (CFI = 1.000, TLI = 1.006, RMSEA = 0.000, and SRMR = 0.027) with good reliability (α = 0.82); furthermore, the PCIBS was negatively and significantly related to a measure of fear of COVID-19 (Chang et al., 2020b). Another study has also reported moderate relationships between PCIBS and psychological distress in an Iranian sample (Ahorsu et al., 2020a).

So far, the psychometric properties of the PCIBS have only been studied in the Taiwanese context, and measurement invariance (MI) across different countries, let alone Latin America, has not been explored. Examining behavioral measures in different samples is useful to identify which aspects have universal utility and which may be applicable only to certain groups (Miller and Sheu, 2009). Without this type of evaluation, the applicability of the measure in cross-cultural studies would be unknown and there would be uncertainty as to whether the findings could be due to true differences in the behavior of interest or measurement error due to using a measure that does not have adequate psychometric properties in all cultural contexts (Caycho, 2017). In this sense, MI is important in between-group comparison studies because it demonstrates whether group members interpret instrument items in the same way (Vandenberg and Lance, 2000). In addition, MI allows us to compare the means of a measure and its correlations with other variables between different groups in a meaningful way (Cheung and Rensvold, 2002).

Traditionally, MI is examined using multigroup confirmatory factor analysis (MG-CFA; Millsap, 2011), where a sequence of increasingly restricted factor models are compared with other less restricted models (Vandenberg and Lance, 2000; Meredith and Teresi, 2006): (1) configural invariance, which evaluates whether the same factor structure (with the same pattern of fixed and free factor loadings) was estimated simultaneously in all groups, without establishing restrictions; (2) metric invariance, where the factor loadings of the items are equal across groups; and (3) scalar invariance, where both factor loadings and intercepts are equal across groups. Metric invariance is necessary to compare covariance or unstandardized regression coefficients across groups. This would indicate that the construct has the same metric and significance in all groups compared. Also, scalar invariance allows for comparison of the means of the construct, indicating that the measure is used in the same way across groups (Byrne et al., 1989). However, some suggest that the full scalar invariance model, based on the MG-CFA, is an ideal that can practically only be approximated (Marsh et al., 2018). If the results do not suggest the presence of scalar invariance, the presence of partial MI (Byrne et al., 1989) is tested through successive removal of constraints on item intercepts.

The presence of strict invariance is also suggested where the residual variances are equal and indicate that the systematic measurement error is equivalent across groups for similar items (Meredith, 1993). However, in recent years there is some debate regarding the appropriate level of MI for a measurement instrument (Lorenzo-Seva et al., 2019). While it has been suggested that strict invariance is the most complete form and a necessary condition for fair comparisons (Meredith, 1993; Millsap and Meredith, 2007), it often represents an unattainable ideal in applied research and if used as a mandatory standard to achieve it may generate biased parameter estimates (Little, 1997). Therefore, in practice, scalar invariance would be sufficient evidence of MI (Marsh, 1994; Little, 1997; McArdle, 1998; Vandenberg and Lance, 2000).

While MG-CFA is the most widely used method, some suggest that it is a complex and impractical method when a large number of groups need to be compared (Asparouhov and Muthén, 2014). Also, fit indices, such as Chi-square, comparative fit index (CFI) or root mean square error of approximation (RMSEA) may not work properly when comparing multiple groups, which would lead to modifications to improve the model, and have a higher probability of model misspecification, which would mean that the final model is not replicable (Asparouhov and Muthén, 2014). In this sense, the traditional approach is too strict as it rejects models that are practically comparable across groups (Lomazzi, 2018). Moreover, it is often impossible to achieve invariance as the possible violations in terms of strict equivalence increase as the number of groups increases (Davidov et al., 2014).

To overcome these limitations, in addition to the MG-CFA, an alternative method has been used in this study: the Multi-Group Factor Analysis Alignment (CFA-MIAL; Asparouhov and Muthén, 2014). The CFA-MIAL is a viable alternative to traditional models which allows for automating and simplifying, to a large extent, MI (Marsh et al., 2018). The CFA-MIAL enables estimating the mean of the factors and variance parameters in each group in order to minimize the amount of non-invariance, thus, allowing the items to have a minimum difference in the parameters of the factor loadings and intercepts. In this sense, unlike the MG-CFA, which tests levels of MI step by step, the CFA-MIAL, evaluates the invariance of factor loadings and intercepts simultaneously (Asparouhov and Muthén, 2014). Also, the CFA-MIAL can be useful for comparing latent means even when there is insufficient evidence of complete scalar invariance. However, it is important to specify that the starting point of the CFA-MIAL lies in the typical GM-CFA tests: invariance of factor loadings, intercepts and latent means (Marsh et al., 2009; Millsap, 2011). Thus, if there is initially sufficient evidence to support scalar invariance, there would be no need to use the CFA-MIAL (Marsh et al., 2018).

Additionally, the properties of the PCIBS were originally examined on the basis of classical test theory (CTT), which considers a measure as an integrated whole rather than at the item level. CTT is based on the assumption that each person possesses an inherent attribute, expressed in the true score, which is made up of the observed score and random error. In this sense, the smaller the error variance, the more accurately the true scores (or inherent attributes) are reflected in the observed scores (Crocker and Algina, 1991). However, the item response theory (IRT) model has advantages over CTT and has been used for the evaluation of the psychometric properties of instruments measuring mental health indicators during the COVID-19 pandemic in several Latin American countries (see Caycho-Rodríguez et al., 2020, 2021a, b, c). IRT allows for establishing a relationship between item properties, individuals’ responses to these items, and the underlying trait being measured (Steinberg and Thissen, 2013). In addition, the IRT model provides information about the difficulty and discrimination properties of the items, which can give evidence for the accuracy of the measure (Van der Linden and Hambleton, 1997). Although CTT remains the predominant method in psychometric assessment, the use of IRT is increasing (Xu et al., 2020).

Although the PCIBS has been used in different studies (see Ahorsu et al., 2020b; Chang et al., 2020b), its psychometric properties have not been evaluated across different countries. Therefore, the present study aimed to evaluate the cross-cultural MI of the PCIBS in a large sample of the general population from 12 Latin American countries. The evaluation included an analysis of the unidimensionality of the PCIBS, its reliability and cross-cultural MI based on CTT (in the case of MI, the CFA-MIAL and MG-CFA methods were used); in addition, item difficulty and discrimination parameters were estimated, as well as information functions using IRT. Similarly, the means of the PCIBS were compared among the 12 participating countries. Because different methods (such as CTT and IRT or CFA-MIAL and MG-CFA) are based on different techniques and may generate different results, few studies, such as this one, present a direct comparison between them during the psychometric evaluation of a measurement instrument. More psychometric information, derived from the different methods, will allow for a higher quality instrument for more systematic monitoring as well as facilitate the evaluation of factors influencing changes in preventive behavior during the COVID-19 pandemic in Latin America.

## Materials and Methods

### Design

This study used an instrumental design that evaluates the psychometric properties of psychological measurement instruments (Ato et al., 2013).

### Participants

A total of 5183 people from 12 Latin American countries (Argentina, Bolivia, Chile, Colombia, Cuba, Ecuador, El Salvador, Guatemala, Mexico, Paraguay, Peru, and Uruguay) participated. The snowball sampling method was used for the inclusion of participants and they were encouraged to send the online survey to their own contacts as much as possible. Participants were included if they were 18 years of age or older and provided informed consent. The minimum number of participants in each country was calculated using Soper software (2021). For this, we considered 5 observed variables, corresponding to the 5 items, 1 latent variable, an anticipated effect size (λ = 0.3), probability (α = 0.05) and statistical power (1 – β = 0.95). The software indicated a minimum number of 100 participants per country; however, all participating countries exceeded the minimum number required.

For our sample, the highest average age was recorded for participants from Argentina (*M* = 44; *SD* = 16.2 years) and Guatemala (*M* = 41.6; *SD* = 12.2 years); while the lowest average age was found in the participants from Cuba (*M* = 25.1; *SD* = 7.3 years) and Ecuador (*M* = 29.1; *SD* = 10.6 years). It can also be observed that in every country there is a higher proportion of women (>60%), from 62.8% in Cuba and El Salvador to 79.1% in Uruguay. Similarly, in most countries, the highest percentage of participants were single (from 44.3% in Argentina to 72.6% in El Salvador); however, in Guatemala, the largest group were married (45.1%). Regarding educational level, the highest percentage of participants in every country had completed university studies (from 29.1% in El Salvador to 65.3% in Chile). Likewise, the sample in every country was predominantly urban (>74%), ranging from 74.9% in Ecuador to 96.6% in Uruguay. Additionally, in most countries, participants had a permanent salaried job (from 41.7% in Peru to 71.6% in Cuba); however, in Colombia and Ecuador most people were unemployed at the time of participating in the study (50.5 and 46.8% respectively). In addition, it is worth noting that the majority of participants in every country reported that they had not had COVID-19 (from 49.9% in El Salvador to 86.8% in Cuba). However, in most countries the majority of participants reported that they had had family members with COVID-19 as in the case of Argentina (52%), Bolivia (77.8%), Colombia (64.2%), Ecuador (54.4%), El Salvador (51%), Guatemala (58.6%), Mexico (69.3%), Paraguay (52%), and Peru (67.5%); while in the remaining countries, the majority of participant’s family members had not had the disease. Finally, in almost every country it was reported that the participants had friends infected with COVID-19 (>56%), except in Uruguay where 60.3% of the participants indicated that they had no friends infected with the disease. Further details of the sociodemographic characteristics of the participants can be seen in Table 1.

**TABLE 1:** Sociodemographic characteristics of participants in the Americas.

Socio-demographic data	Argentina (*n* = 325)	Bolivia (*n* = 252)	Chile (*n* = 524)	Colombia (*n* = 372)	Cuba (*n* = 317)	Ecuador (*n* = 451)
Age (M ± SD)	44 ± 16.2	39.3 ± 14.5	36.4 ± 12	30.5 ± 13.1	25.1 ± 7.3	29.1 ± 10.6
**Gender, *n* (%)**						
Male	76 (23.4%)	75 (29.8%)	124 (23.7%)	101 (27.2%)	118 (37.2%)	137 (30.4%)
Female	249 (76.6%)	177 (70.2%)	400 (76.3%)	271 (72.8%)	199 (62.8%)	314 (69.6%)
**Marital status, *n* (%)**						
Single	144 (44.3%)	125 (49.6%)	257 (49%)	267 (71.8%)	205 (64.7%)	309 (68.5%)
Married	95 (29.2%)	82 (32.5%)	142 (27.1%)	59 (15.9%)	48 (15.1%)	92 (20.4%)
Divorced	30 (9.2%)	31 (12.3%)	41 (7.8%)	13 (3.5%)	13 (4.1%)	32 (7.1%)
Living together	41 (12.6%)	9 (3.6%)	80 (15.3%)	28 (7.5%)	50 (15.8%)	14 (3.1%)
Widowed	15 (4.6%)	5 (2%)	4 (0.8%)	5 (1.3%)	1 (0.3%)	4 (0.9%)
**Educational level, *n* (%)**						
Primary incomplete	0 (0%)	0 (0%)	0 (0%)	0 (0%)	0 (0%)	0 (0%)
Primary complete	3 (0.9%)	1 (0.4%)	1 (0.2%)	1 (0.3%)	0 (0%)	1 (0.1%)
Secondary incomplete	5 (1.5%)	5 (2%)	3 (0.6%)	8 (2.2%)	4 (0.9%)	5 (1.1%)
Secondary complete	31 (9.5%)	7 (2.8%)	21 (4%)	64 (17.2%)	5 (1.6%)	72 (16%)
Technical school incomplete	4 (1.2%)	0 (0%)	8 (1.5%)	7 (1.9%)	1 (0.3%)	4 (0.9%)
Technical school complete	32 (9.8%)	15 (6%)	43 (8.2%)	36 (9.7%)	10 (3.2%)	11 (2.4%)
University incomplete	84 (25.8%)	60 (23.8%)	106 (20.2%)	118 (31.7%)	150 (47.3%)	130 (28.8%)
University complete	166 (51.1%)	164 (65.1%)	342 (65.3%)	138 (37.1%)	148 (46.7%)	228 (50.6%)
**Type of work, *n* (%)**						
Fixed job	207 (63.7%)	106 (42.1%)	300 (57.3%)	188 (31.7%)	227 (71.6%)	170 (37.7%)
Temporary job	41 (12.6%)	59 (23.4%)	77 (14.7%)	66 (17.7%)	14 (4.4%)	70 (15.5%)
Unemployed	77 (23.7%)	87 (34.5%)	147 (28.1%)	188 (50.5%)	76 (24%)	211 (46.8%)
Area of residence, *n* (%)						
Urban	309 (95.1%)	243 (96.4%)	452 (86.3%)	344 (92.5%)	278 (87.7%)	338 (74.9%)
Rural	16 (4.9%)	9 (3.6%)	72 (13.7%)	28 (7.5%)	39 (12.3%)	113 (25.1%)
**Has had COVID-19, *n* (%)**						
Yes	50 (15.4%)	73 (29%)	30 (5.7%)	69 (18.5%)	5 (1.6%)	73 (16.2%)
No	216 (66.5%)	137 (54.4%)	437 (83.4%)	211 (56.7%)	275 (86.8%)	286 (63.4%)
I don’t know, but I think so	19 (5.8%)	28 (11.1%)	15 (2.9%)	53 (14.2%)	9 (2.8%)	52 (11.5%)
I don’t know, but I think not	40 (12.3%)	14 (5.6%)	42 (8%)	39 (10.5%)	28 (8.8%)	40 (8.9%)
**Family with COVID-19, *n* (%)**						
Yes	169 (52%)	196 (77.8%)	227 (43.3%)	239 (64.2%)	77 (24.3%)	244 (54.4%)
No	156 (48%)	56 (22.2%)	297 (56.7%)	133 (35.8%)	240 (75.7%)	207 (45.9%)
**Friends with COVID-19, *n* (%)**						
Yes	284 (87.4%)	241 (95.6%)	335 (63.9%)	306 (82.3%)	179 (56.5%)	375 (83.1%)
No	41 (12.6%)	11 (4.4)	189 (36.1%)	66 (17.7%)	138 (43.5%)	76 (16.9%)

**Socio-demographic data**	**El Salvador (*n* = 698)**	**Guatemala (*n* = 324)**	**México (*n* = 300)**	**Paraguay (*n* = 877)**	**Perú (*n* = 360)**	**Uruguay (*n* = 383)**

Age (M ± SD)	29.4 ± 8.9	41.6 ± 12.2	33.6 ± 13.7	31.5 ± 10.9	31.8 ± 10.9	38.9 ± 14.3
**Gender, *n* (%)**						
Male	260 (37.2%)	114 (35.2%)	98 (32.7%)	212 (24.2%)	114 (31.7%)	80 (20.9%)
Female	438 (62.8%)	210 (64.8%)	202 (67.3%)	665 (75.8%)	246 (68.3%)	303 (79.1%)
**Marital status, *n* (%)**						
Single	507 (72.6%)	126 (28.9%)	168 (56%)	577 (65.8%)	231 (64.2%)	171 (44.6%)
Married	127 (18.2%)	146 (45.1%)	95 (31.7%)	202 (23%)	69 (19.2%)	87 (22.7%)
Divorced	11 (1.6%)	26 (8%)	18 (6%)	25 (2.9%)	17 (4.7%)	43 (11.2%)
Living together	51 (7.3%)	20 (6.2%)	14 (4.7%)	67 (7.6%)	41 (11.4%)	75 (19.6%)
Widowed	2 (0.3%)	6 (1.9%)	5 (1.7%)	6 (0.7%)	2 (0.6%)	7 (1.8%)
**Educational level, *n* (%)**						
Primary incomplete	13 (1.9%)	0 (0%)	0 (0%)	0 (0%)	1 (0.1%)	0 (0%)
Primary complete	10 (1.4%)	1 (0.3%)	0 (0%)	4 (0.5%)	0 (0%)	1 (0.3%)
Secondary incomplete	48 (6.9%)	10 (3.1%)	1 (0.3%)	17 (1.9%)	4 (1.1%)	24 (6.3%)

**Socio-demographic data**	**Argentina (*n* = 325)**	**Bolivia (*n* = 252)**	**Chile (*n* = 524)**	**Colombia (*n* = 372)**	**Cuba (*n* = 317)**	**Ecuador (*n* = 451)**

Secondary complete	106 (15.2%)	21 (6.5%)	20 (6.7%)	76 (8.7%)	14 (3.9%)	36 (9.4%)
Technical school incomplete	8 (1.1%)	6 (1.9%)	4 (1.3%)	2 (0.2%)	7 (1.9%)	1 (0.3%)
Technical school complete	31 (4.4%)	18 (5.6%)	42 (14%)	20 (2.3%)	21 (5.8%)	38 (9.9%)
University incomplete	279 (40%)	67 (20.7%)	82 (27.3%)	292 (33.3%)	105 (29.2%)	113 (29.5%)
University complete	203 (29.1%)	201 (62%)	151 (50.3%)	466 (53.1%)	208 (57.8%)	170 (44.4%)
**Type of work, *n* (%)**						
Fixed job	370 (53%)	223 (68.8%)	142 (47.3%)	487 (55.5%)	150 (41.7%)	268 (70%)
Temporary job	86 (12.3%)	45 (13.9%)	57 (19%)	149 (17%)	77 (21.4%)	24 (6.3%)
Unemployed	242 (34.7%)	56 (17.3%)	101 (33.7%)	241 (27.5%)	133 (36.9%)	91 (23.8%)
**Area of residence, *n* (%)**						
Urban	550 (78.8%)	304 (93.8%)	279 (93%)	774 (88.3%)	318 (88.3%)	370 (96.6%)
Rural	148 (21.2%)	20 (6.2%)	21 (7%)	103 (11.7%)	42 (11.7%)	13 (3.4%)
**Has had COVID-19, *n* (%)**						
Yes	113 (16.2%)	28 (8.6%)	47 (15.7%)	132 (15.1%)	74 (20.6%)	10 (2.6%)
No	348 (49.9%)	256 (79%)	196 (65.3%)	556 (63.4%)	205 (56.9%)	320 (83.6%)
I don’t know, but I think so	177 (25.4%)	24 (7.4%)	27 (9%)	94 (10.7%)	53 (14.7%)	5 (1.3%)
I don’t know, but I think not	60 (8.6%)	16 (4.9%)	30 (10%)	95 (10.8%)	28 (7.8%)	48 (12.5%)
**Family with COVID-19, *n* (%)**						
Yes	356 (51%)	190 (58.6%)	208 (69.3%)	465 (53%)	243 (67.5%)	81 (21.1%)
No	342 (49%)	134 (41.4%)	92 (30.7%)	412 (47%)	117 (32.5%)	302 (78.9%)
**Friends with COVID-19, *n* (%)**						
Yes	514 (73.6%)	289 (89.2%)	252 (84%)	702 (80%)	310 (86.1%)	152 (39.7%)
No	184 (26.4%)	35 (10.8%)	48 (16%)	175 (20%)	50 (13.9%)	231 (60.3%)

### Instruments

#### Preventive COVID-19 Infection Behaviors Scale

The PCIBS (Chang et al., 2020b) assesses the frequency with which individuals engage in COVID-19 preventive behaviors. It consists of five items that measure five preventive behaviors recommended by the WHO. Each item is scored from 1 (almost never) to 5 (almost always), where the total score is calculated by averaging the responses to all items. Higher scores indicate that people engage in COVID-19 preventive behaviors more frequently. The PCIBS was translated based on World Health Organization [WHO] (2020) suggestions for instrument translation and adaptation. First, the PCIBS was translated from English to Spanish by a bilingual expert whose native language was English. Second, another bilingual professional (a native Spanish speaker) translated this initial Spanish version back into English. Third, a 4-member panel of experts evaluated the translations. Fourth, the PCIBS was administered to a focus group sample of 20 people (12 women and 8 men, mean age = 25.76) in order to have an initial evaluation of the scale, as well as to determine the time needed and possible difficulties in answering the questions. Participants could suggest any changes they felt were necessary. No changes were made because the focus group sample indicated that they were not necessary. Table 2 presents the original English version and the Spanish version used in this study.

**TABLE 2:** Original English version of the PCIBS and the translation into Spanish.

Items from the original English version	Translation of the Items in the Spanish version
Item 1: How often do you regularly and thoroughly clean your hands with an alcohol-based hand rub or wash them with soap and water?	Item 1: ¿Con qué frecuencia se lava las manos de forma regular y completa con un desinfectante para manos a base de alcohol o con agua y jabón?
Item 2: How often do you avoid touching eyes, nose, and mouth?	Item 2: ¿Con qué frecuencia evita tocarse los ojos, la nariz y la boca?
Item 3: How often do you cover your mouth and nose with your bent elbow or tissue when you cough or sneeze?	Item 3: ¿Con qué frecuencia se tapa la boca y la nariz con el brazo o con un pañuelo cuando tose o estornuda?
Item 4: How often do you maintain at least 1-m distance between yourself and others?	Item 4: ¿Con qué frecuencia mantienes al menos 1 metro de distancia entre tú y los demás?
Item 5: How often do you stay home when you feel unwell?	Item 5: ¿Con qué frecuencia te quedas en casa cuando no te sientes bien?

*This translation followed a process of forward and back translation with a focus group to review the translated Spanish version.*

#### Coronavirus Anxiety Scale

The Coronavirus Anxiety Scale (CAS, Lee, 2020) is a unidimensional measure that assesses physiological reactions to anxiety related to COVID-19. In this study we used the Spanish version (Caycho-Rodríguez et al., 2021b) that has been cross-culturally validated for 12 Latin American countries (Argentina, Bolivia, Chile, Colombia, Cuba, Ecuador, El Salvador, Guatemala, Mexico, Paraguay, Peru, and Uruguay) (Caycho-Rodríguez et al., 2021d) consisting of four items (e.g., “I felt dizzy, lightheaded, or faint when I read or listened to news about the coronavirus”). With five Likert-type response options (from 0 = not at all to 4 = almost every day during the last 2 weeks). Each of the four items is more informative for average and high levels of COVID-19 dysfunctional anxiety than at lower levels.

### Procedure

This study was part of a larger project to cross-culturally validate brief measures of mental health indicators during the COVID-19 pandemic in Latin America (see for example, Caycho-Rodríguez et al., 2021e). The study was conducted between February 17 and March 17, 2021. In this period of time, the participating countries experienced different phases of the COVID-19 pandemic. In Uruguay, there were about 10,923 diagnosed cases and 15 deaths, with a weekly average of 9. Thus, Uruguay went from being in a situation of low infection during 2020 to an increase of 33.51 on average per 100,000 people. In El Salvador, there were 63,344 confirmed cases of COVID-19, of which 1,986 were reported as deaths and 60,681 recovered. Regarding the phase of the disease, the type of transmission in El Salvador was classified by the WHO as “Local,” specifically at the community level. In the case of Argentina, an average of 6962 diagnosed cases and 125 deaths were reported in the last 7 days. In Cuba, the country was in a phase of resurgence (second wave) characterized by daily diagnoses of high numbers of people infected with the virus, much higher compared to diagnoses on a similar date in the first stage of the disease. During the period of application of the survey in the country, 2,730,305 samples had been studied, of which 64,414 were positive; 3,596 were active cases, 60,378 patients recovered and 384 patients passed away. There were 1,039,623 positive cases in Chile (896,231 with laboratory confirmation and 143,392 probable cases without laboratory confirmation) with a cumulative incidence rate of 5,342.8 per 100,000 populations. In addition, a cumulative number of 21,674 deaths were recorded, with the country being in the second great wave of contagion and in the middle of the initial stage of the massive vaccination campaign that began on February 3, 2021. In Ecuador, 16,780 deaths were reported due to COVID-19, as the country faced one of the stages with the highest rates of hospitalization due to COVID-19; while in Guatemala, there were more than 164,746 confirmed cases and 5,989 deaths. In Bolivia, during the months of February and March 2021, about 272,411 cases of infection and 4,538 deaths were confirmed; in addition, vaccination against COVID-19 was initiated in early March 2021. Colombia was at the end of the second wave of infection in the country, which resulted in a decrease in the number of infections and deaths. Thus, 106,453 new infections, 32,264 active cases and 3,356 deaths were recorded in the country. In addition, this period was characterized by economic reopening in most sectors, except for entertainment (cinemas and theaters), sporting events which had no audiences in the stands, and social gatherings with a maximum capacity of 50 people. In Mexico, during the study period, there were more than 2,238,887 people infected and 203,210 total deaths due to COVID-19, giving a daily average of 560 deaths associated with COVID-19. In Paraguay, the total number of confirmed cases rose from 139,819 to 157,603, the number of active cases rose from 20,897 to 22,990 cases, and the number of deaths rose from 2,862 to 3,152. Finally, in Peru, up to March 2021, 1,533,121 cases were confirmed, 15,497 patients were hospitalized for COVID-19, of whom 2,278 were in an ICU with mechanical ventilation, and 51,635 deaths from the disease were reported.

All participating countries followed the same data collection procedure. An online questionnaire was developed, using the Google Form platform, which was distributed through social networks (Facebook, Twitter, and WhatsApp) and emails. The online questionnaire had an introductory section where there was information about the aim of the study, informed consent information and contact information in case participants had any questions about the research. All participants gave informed consent and were guaranteed confidentiality of their data and the freedom to withdraw from the study at any time. The study followed the recommendations of the Declaration of Helsinki and was approved by the ethics committee of the Universidad Privada del Norte in Peru (registry number: 20213002).

### Data Analysis

For the Confirmatory Factor Analysis (CFA), the *Diagonally Weighted Least Squares with Mean and Variance corrected* (WLSMV) estimator was used since the items are at the ordinal level (Brown, 2015). To assess the model fit, the chi-square test (χ2), the RMSEA index and the SRMR index were used in which case values less than 0.05 indicate good fit, and between 0.05 and 0.08 is considered acceptable (Kline, 2015). In addition, the CFI and TLI index were used, where values greater than 0.95 indicate good fit and greater than 0.90 an acceptable fit (Schumacker and Lomax, 2015). To evaluate the internal consistency of the scale, *Cronbach’s alpha* coefficient (Cronbach, 1951) and the omega coefficient (McDonald, 1999) were used, where a value greater than 0.70 is adequate (Viladrich et al., 2017).

To evaluate the factorial invariance of the scale according to the nationality of the participants (country), two methodological approaches were used: (a) exact MI (traditional approach) and (b) approximate measurement invariance (AMI). Both approaches are based on different assumptions: In the traditional approach, factor weights and intercepts must be exactly equal across groups to evidence invariance. In contrast, the second approach considers that factor weights and intercepts do not have to be identical between groups that are culturally different and therefore some small differences in parameters can be accepted (Byrne and van de Vijver, 2017; Lomazzi, 2018; Fischer and Karl, 2019).

Under the traditional approach, Multi-group Confirmatory Factor Analysis (MG-CFA) was used, where a sequence of hierarchical invariance models was proposed. First, configural invariance (reference model) was evaluated, followed by metric invariance (equality of factor loadings) and scalar invariance (equality of factor loadings and intercepts). When scalar invariance was not found, modification rates and expected parameter change were assessed (Whittaker, 2012). This allowed us to identify and release some parameters so that they can vary between groups and with this re-specified model test for partial invariance (Beaujean, 2014).

To compare the sequence of models we first used a formal statistical test, for which we used the chi-square difference (Δχ2) where non-significant values (*p* > 0.05) suggest invariance between groups. Secondly, a modeling strategy was employed, for which the differences in CFI (ΔCFI) was used, where values less than <0.010 evidence model invariance between groups (Chen, 2007). The change in RMSEA (ΔRMSEA) was also used, where differences less than <0.015 show the invariance of the model between groups (Chen, 2007).

Regarding the AMI, the Multi-Group Factor Analysis Alignment (CFA-MIAL) was used to test for invariance (Asparouhov and Muthén, 2014). This method was performed in two steps: In the first stage, an unconstrained configural model was fitted across all groups. In the second stage, this configural model was optimized using a component loss function with the aim of minimizing the invariance in factor means and factor variances for each group (Asparouhov and Muthén, 2014). At this stage, the invariance tolerance criteria for factor weights (λ = 0.40) and intercepts (ν = 0.20) were established according to the recommendations of Robitzsch (2020). In addition, alignment power was set to 0.25 for both parameters (Fischer and Karl, 2019). To assess the invariance of the parameters, the R2 index was assessed. Values close to 1 indicate a high degree of invariance, while values close to 0 indicate a low degree of invariance (Asparouhov and Muthén, 2014). To assess the percentage of non-invariant parameters (λ and ν), a cut-off of 25% was set to consider a scale as non-invariant (Asparouhov and Muthén, 2014). To examine differences between countries in a convenient way, composite scores were created by summing the final scale items. Cohen’s d test was used to assess the magnitude of differences.

For Item Response Theory (IRT), a Graded Response Model (GRM, Samejima, 1997) was used, specifically an extension of the 2-parameter logistic model (2-PLM) for ordered polytomous items (Hambleton et al., 2010). The C2 test developed for ordinal items (Cai and Monroe, 2014) was used to estimate the model fit and the following fit criteria were used: RMSEA ≤ 0.05 (Maydeu-Olivares and Joe, 2014) and SRMSR ≤ 0.05 (Maydeu-Olivares, 2013). CFI and TLI values were also taken into account using the same fit criterion (≥0.95) employed in SEM models (Lubbe and Schuster, 2019). For each item, two types of parameters were estimated: discrimination (a) and difficulty (b). The discrimination parameter (a) determines the slope at which item responses change as a function of the level of the latent trait and the item difficulty parameters (b) determine how much of the latent trait the item requires to be answered in a given way. As the scale has four response categories, there are three difficulty estimates, one per threshold. The estimates for these three thresholds indicate the level of the latent variable at which an individual has a 50% chance of scoring at or above a particular response category. Information Curves were also calculated for the items and the scale (IIC and TIC respectively).

Regarding the evidence of validity in relation to other variables, an SEM model was used. In this model, COVID-19 preventive behaviors were related to COVID-19 anxiety. The WLSMV estimator was used to estimate the model and the same adjustment indicators used in the CFA were taken into account.

All statistical analyses were performed using the “lavaan” package (Rosseel, 2012) for the CFA, the “semTools” package (Jorgensen et al., 2018) for factorial invariance, the “sirt” package (Robitzsch, 2020) for the Alignment method and the “mirt” package for the GRM (Chalmers, 2012). In all cases, the RStudio environment for R was used (R Core Team, 2019).

## Results

### Descriptive Analysis

Table 3 shows that item 3 (“How often do you cover your mouth and nose with your arm or handkerchief when you cough or sneeze?”) has the highest average score in all countries except Guatemala. It can also be seen that most of the items in the polychoric correlation matrix have a moderate to high correlation coefficient. However, item 5 has a low correlation coefficient with the other items of the scale. This pattern is evident in all countries. With respect to the skewness and kurtosis indices, it can be seen that the items present adequate indices in most of the countries (As < ± 2; Ku < ± 7) according to the criteria of Finney and DiStefano (2006). However, item 3 presents a markedly asymmetric response pattern in the countries of Mexico (As = –2.33), Bolivia (As = –2.31), and Chile (As = –2.22).

**TABLE 3:** Descriptive analysis of the items and polychoric correlation matrix.

Region – country	Items	*M*	*SD*	*g1*	*g2*	Polychoric correlation matrix				
						1	2	3	4	5
Mexico (*n* = 300)	1	3.37	0.96	–1.81	3.12	1				
	2	2.77	1.33	–0.65	–0.93	0.62	1			
	3	3.58	0.94	–2.33	4.67	0.73	0.57	1		
	4	3.34	1.07	–1.63	1.81	0.66	0.56	0.73	1	
	5	2.99	1.34	–1.03	–0.32	0.38	0.31	0.52	0.49	1
Guatemala (*n* = 324)	1	3.40	0.87	–1.62	2.56	1				
	2	2.78	1.25	–0.63	–0.88	0.53	1			
	3	3.39	1.02	–1.51	1.07	0.41	0.50	1		
	4	3.25	0.99	–1.21	0.69	0.48	0.43	0.39	1	
	5	2.53	1.38	−38	–1.21	0.23	0.25	0.34	0.34	1
El Salvador (*n* = 698)	1	3.27	0.99	–1.38	1.26	1				
	2	2.81	1.25	–0.66	–0.81	0.55	1			
	3	3.36	1.01	–1.54	1.54	0.51	0.59	1		
	4	3.07	1.13	–0.92	–0.31	0.40	0.46	0.50	1	
	5	2.52	1.41	–0.40	–1.22	0.18	0.20	0.25	0.44	1
Cuba (*n* = 317)	1	3.42	0.86	–1.78	3.59	1				
	2	2.96	1.25	–0.86	–0.54	0.57	1			
	3	3.42	1.06	–1.76	1.97	0.31	0.35	1		
	4	2.99	1.18	–0.78	–0.63	0.38	0.46	0.39	1	
	5	2.24	1.45	–0.06	–1.42	0.20	0.22	0.23	0.35	1
Peru (*n* = 360)	1	3.33	0.95	–1.52	1.87	1				
	2	2.73	1.24	–0.55	–0.93	0.56	1			
	3	3.44	1.00	–1.79	2.20	0.50	0.52	1		
	4	3.33	0.96	–1.44	1.46	0.51	0.59	0.48	1	
	5	2.98	1.26	–0.95	–0.39	0.20	0.27	0.35	0.32	1
Bolivia (*n* = 252)	1	3.44	0.87	–1.74	2.91	1				
	2	2.78	1.25	–0.60	–0.92	0.60	1			
	3	3.59	0.88	–2.31	4.85	0.47	0.46	1		
	4	3.26	0.94	–1.09	0.26	0.33	0.32	0.32	1	
	5	2.47	1.45	–0.32	−139	0.08	0.14	0.31	0.28	1
Ecuador (*n* = 451)	1	3.25	0.97	–1.38	1.64	1				
	2	2.77	1.19	–0.59	–0.76	0.56	1			
	3	3.41	1.00	–1.77	2.43	0.54	0.52	1		
	4	3.13	1.03	–1.02	0.23	0.50	0.52	0.60	1	
	5	2.69	1.29	–0.54	–0.93	0.29	0.35	0.41	0.46	1
Colombia (*n* = 372)	1	3.23	0.99	–1.48	1.99	1				
	2	2.62	1.22	–0.36	–1.05	0.56	1			
	3	3.31	1.05	–1.45	1.16	0.39	0.48	1		
	4	3.06	1.09	–0.97	0.03	0.50	0.42	0.48	1	
	5	2.90	1.28	–0.83	–0.56	0.23	0.31	0.34	0.34	1
Chile (*n* = 524)	1	3.26	0.97	–1.30	1.18	1				
	2	2.54	1.24	–0.26	–1.13	0.55	1			
	3	3.58	0.87	–2.22	4.42	0.47	0.43	1		
	4	3.33	0.94	–1.40	1.39	0.44	0.38	0.51	1	
	5	2.73	1.41	–0.64	–1.02	0.10	0.15	0.22	0.24	1
Argentina (*n* = 325)	1	3.25	0.97	–1.43	1.72	1				
	2	2.43	1.34	–0.27	–1.21	0.64	1			
	3	3.49	0.99	–2.03	3.28	0.53	0.58	1		
	4	3.14	1.11	–1.13	0.30	0.40	0.33	0.43	1	
	5	2.44	1.39	–0.29	–1.29	0.24	0.25	0.39	0.30	1
Uruguay (*n* = 383)	1	3.23	1.01	–1.32	1.19	1				
	2	2.15	1.36	0.01	–1.23	0.60	1			
	3	3.27	1.02	–1.43	1.03	0.37	0.41	1		
	4	2.97	1.09	–0.73	–0.44	0.35	0.46	0.30	1	
	5	2.46	1.42	–0.29	–1.33	0.22	0.30	0.23	0.40	1
Paraguay (*n* = 877)	1	3.27	0.96	–1.49	2.06	1				
	2	2.48	1.31	–0.27	–1.21	0.59	1			
	3	3.39	1.03	–1.62	1.59	0.52	0.52	1		
	4	2.97	1.14	–0.77	–0.56	0.44	0.41	0.45	1	
	5	2.46	1.37	–0.26	–1.28	0.21	0.24	0.33	0.33	1

*M, mean; SD, standard deviation; g1, skewness; g2, kurtosis.*

### Validity Based on Internal Structure and Reliability of the Scale

Table 4 shows that the original five-item model (model 1) does not show adequate adjustment indexes in most of the countries, especially in El Salvador, Bolivia, Colombia, Argentina, Uruguay, and Paraguay. In addition, it can be seen that the factorial weight of item 5, unlike the other items, is low in almost all the countries. Taking this into account, item 5 (“How often do you stay at home when you feel unwell?”) was removed and a model of four items was evaluated (model 2). It can be seen in Table 3 that this model presents excellent fit indices in all countries, except in Chile and Colombia, where it obtained acceptable indices. In addition, all the items in this second model present a factorial weight between moderate and high in all the countries. Therefore, Model 2 was used for the remaining analyses.

**TABLE 4:** Fit indices, factorial weights, and reliability of the unidimensional models in American countries.

Model	Country	χ ^2^	df	*p*	CFI	TLI	SRMR	RMSEA [90%CI]	Factorial weight					Reliability	
									1	2	3	4	5	α	ω
1	Mexico (9)	10.05	5	0.074	0.99	0.99	0.031	0.058 [0.000 –0.110]	0.82	0.69	0.88	0.83	0.53	0.86	0.79
	Guatemala (8)	10.13	5	0.072	0.99	0.98	0.039	0.056 [0.000 –0.107]	0.69	0.72	0.65	0.65	0.42	0.76	0.68
	El Salvador (7)	75.05	5	0.000	0.94	0.88	0.061	0.142 [0.114 –0.171]	0.67	0.75	0.77	0.67	0.40	0.78	0.73
	Cuba (5)	16.27	5	0.006	0.97	0.93	0.047	0.084 [0.041 –0.132]	0.68	0.75	0.51	0.65	0.39	0.73	0.65
	Peru (11)	9.48	5	0.091	0.99	0.99	0.050	0.050 [0.000 –0.098]	0.70	0.78	0.69	0.74	0.39	0.79	0.73
	Bolivia (2)	21.18	5	0.001	0.95	0.89	0.065	0.114 [0.067 –0.165]	0.75	0.74	0.65	0.48	0.29	0.71	0.62
	Ecuador (6)	19.57	5	0.002	0.99	0.97	0.034	0.080 [0.045 –0.119]	0.70	0.72	0.77	0.77	0.52	0.82	0.77
	Colombia (4)	23.10	5	0.000	0.97	0.93	0.043	0.099 [0.060 –0.141]	0.71	0.72	0.66	0.68	0.44	0.77	0.72
	Chile (3)	19.68	5	0.001	0.98	0.95	0.044	0.075 [0.042 –0.111]	0.73	0.68	0.69	0.65	0.25	0.73	0.64
	Argentina (1)	18.99	5	0.002	0.98	0.95	0.048	0.093 [0.051 –0.139]	0.77	0.77	0.75	0.52	0.40	0.77	0.71
	Uruguay (12)	19.44	5	0.002	0.97	0.94	0.049	0.087 [0.048 –0.129]	0.69	0.81	0.51	0.60	0.44	0.74	0.70
	Paraguay (10)	40.04	5	0.000	0.97	0.95	0.039	0.089 [0.065 –0.116]	0.74	0.73	0.72	0.61	0.39	0.77	0.71

2	Mexico (9)	2.84	2	0.242	0.99	0.99	0.018	0.037 [0.000 –0.127]	0.84	0.70	0.87	0.82	–	0.88	0.81
	Guatemala (8)	2.74	2	0.254	0.99	0.99	0.022	0.034 [0.000 –0.121]	0.71	0.74	0.63	0.62	–	0.77	0.70
	El Salvador (7)	4.67	2	0.097	0.99	0.99	0.015	0.044 [0.000 –0.097]	0.69	0.77	0.77	0.61	–	0.80	0.75
	Cuba (5)	6.24	2	0.044	0.99	0.96	0.035	0.082 [0.012 –0.158]	0.69	0.78	0.50	0.61	–	0.74	0.67
	Peru (11)	0.92	2	0.632	1.00	1.00	0.009	0.000 [0.000 –0.083]	0.72	0.79	0.67	0.73	–	0.82	0.76
	Bolivia (2)	0.89	2	0.640	1.00	1.01	0.015	0.000 [0.000 –0.099]	0.77	0.76	0.62	0.44	–	0.74	0.68
	Ecuador (6)	7.34	2	0.026	0.99	0.98	0.023	0.077 [0.023 –0.140]	0.72	0.73	0.77	0.74	–	0.83	0.78
	Colombia (4)	15.24	2	0.000	0.97	0.92	0.039	0.134 [0.076 –0.200]	0.73	0.73	0.64	0.66	–	0.78	0.73
	Chile (3)	12.80	2	0.002	0.98	0.94	0.038	0.102 [0.054 –0.158]	0.75	0.68	0.68	0.63	–	0.78	0.71
	Argentina (1)	7.06	2	0.029	0.99	0.97	0.033	0.088 [0.024 –0.163]	0.79	0.79	0.73	0.50	–	0.79	0.74
	Uruguay (12)	1.61	2	0.448	1.00	1.00	0.016	0.000 [0.000 –0.095]	0.70	0.85	0.51	0.54	–	0.74	0.71
	Paraguay (10)	6.55	2	0.038	0.99	0.99	0.017	0.051 [0.010 –0.097]	0.76	0.75	0.71	0.59	–	0.79	0.73

*χ^2^, Chi square; df, degrees of freedom; SRMR, Standardized Root Mean Square Residual; TLI, Tucker-Lewis Index; CFI, Comparative Fit Index; RMSEA, Root Mean Square Error of Approximation; α, Cronbach’s Alpha; ω, McDonald’s Omega.*

To evaluate the internal consistency of the scale (model 2), the results of the Confirmatory Factor Analysis (CFA) were used. As can be seen in Table 4, the scale shows adequate reliability indices in every country (α ≥ 0.74; ω ≥ 0.67).

### Factorial Invariance by Country

Following the traditional invariance approach, it can be seen in Table 5 that the factor structure of the scale has shown evidence of metric invariance (ΔCFI = –0.01; ΔRMSEA = 0.00). However, when adding the item intercept equality constraint, the fit worsened markedly, evidencing a lack of scalar invariance (ΔCFI = –0.10; ΔRMSEA = 0.04). To assess partial invariance, it is recommended to examine the modification rates and estimated parameter changes to identify the appropriate model parameters that require release from equality constraints (Byrne et al., 1989; Brown, 2015). After examining the modification indices and the expected parameter change, it was decided to release the intercept of items 1 (“How often do you thoroughly and regularly clean your hands with an alcohol-based hand sanitizer or soap and water?”) and 4 (“How often do you avoid touching your eyes, nose, and mouth?”) so that they could vary across countries. The equality restrictions on the intercepts of these items were released because they showed the most severe violations of invariance compared to the other item intercepts in the model. The change made helped the model to improve its fit indices. Thus, partial scalar invariance (ΔCFI = –0.01; ΔRMSEA = 0.01) of the scale was established across countries.

**TABLE 5:** Unidimensional model fit indices and invariance models by country.

Unidimensional model	χ^2^	df	*p*	SRMR	TLI	CFI	RMSEA	Δχ^2^	Δ df	*p*	Δ CFI	Δ RMSEA
Total sample	56.21	2	0.000	0.021	0.98	0.99	0.072	–	–	–	–	–
**By country**												
Configural	37.10	24	0.043	0.015	0.98	0.99	0.036	–	–	–	–	–
Metric	95.27	57	0.001	0.031	0.98	0.98	0.039	39.82	33	0.193	–0.01	0.00
Scalar	320.79	90	0.000	0.053	0.90	0.88	0.077	86.09	33	0.000	–0.10	0.04
Partial scalar^a^	124.56	68	0.000	0.036	0.97	0.97	0.044	16.08	11	0.138	–0.01	0.01

*χ^2^, chi square; df, degrees of freedom; SRMR, Standardized Root Mean Square Residual; TLI, Tucker–Lewis Index; CFI, Comparative Fit Index; RMSEA, Root Mean Square Error of Approximation; Δχ2, Differences in Chi square; Δdf, Differences in degrees of freedom; ΔRMSEA, Change in Root Mean Square Error of Approximation; ΔCFI, Change in Comparative Fix Index.*

*^a^The intercept of items 1 and 2 was freed.*

Under the AMI approach, the CFA-MIAL method showed that the factor structure of the scale is invariant both for the factor loadings (*R*^2^ = 0.99) and for the intercepts of the items (*R*^2^ = 0.99), as shown in Table 6. With respect to the percentage of non-invariant parameters per country, it is observed that all the factor weights are invariant (0%). Regarding the intercepts, the finding of only two non-invariant parameters shows that, at a general level, the percentage of non-invariant parameters is remarkably low (4.2%).

**TABLE 6:** ML invariance aligent (IA) in the American countries.

Parameters	Items	*Me*	*SD*	Min	Max	Countries												*R* ^2^	%
Factorial weight	Prev1	0.63	0.05	0.58	0.76	1	2	3	4	5	6	7	8	9	10	11	12	0.99	0.0%
	Prev2	0.86	0.11	0.66	0.99	1	2	3	4	5	6	7	8	9	10	11	12		
	Prev3	0.60	0.07	0.44	0.64	1	2	3	4	5	6	7	8	9	10	11	12		
	Prev4	0.61	0.08	0.42	0.67	1	2	3	4	5	6	7	8	9	10	11	12		

Intercept	Prev1	3.26	0.07	3.22	3.47	1	2	3	4	5	6	7	8	9	10	11	(12)	0.99	4.2%
	Prev2	2.62	0.11	2.43	2.79	1	2	3	4	5	6	7	8	9	10	11	12		
	Prev3	3.42	0.08	3.30	3.58	1	2	3	4	5	6	7	8	9	10	11	12		
	Prev4	3.15	0.13	2.81	3.33	1	2	3	4	(5)	6	7	8	9	10	11	12		

*%, percentage of item parameters without invariance. Parentheses indicate that the parameter is not invariant for that specific group (country).*

**FIGURE 1:**
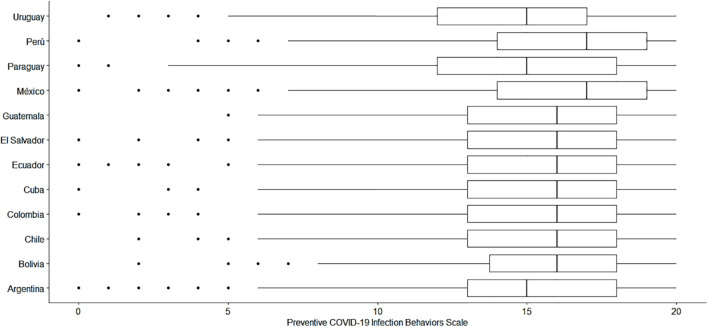
Comparison of the scores of the Preventive COVID-19 Infection Behaviors Scale by country.

Figure 1 shows a graphical representation of the scale scores by country. Most of the differences were irrelevant, although some were relevant but small in size. Among the countries showing a larger difference, Mexico was found to have higher scores than Uruguay (*d* = 0.48). Similarly, Peru has higher scores than Uruguay (*d* = 0.45).

### Item Response Theory Model: Graded Response Model

The results found in the CFA allow the two main assumptions to be met: the existence of unidimensionality and consequently local independence. Therefore, a Graded Response Model (GRM) was used, specifically an extension of the 2-parameter logistic model (2-PLM) for ordered polytomous items. Table 7 shows that the GRM model presents adequate fit indices (M2[df] = 32.07[2]; *p* < 0.01; RMSEA = 0.05; SRMRS = 0.02; TLI = 0.98; CFI = 0.99). It can also be observed that all items have discrimination parameters above the value of 1, generally considered as good discrimination (Hambleton et al., 2010). Regarding the difficulty parameters, all threshold estimators increased monotonically.

**TABLE 7:** Discrimination and difficulty parameters for the scale items.

Model	Item	Parameters of the items					GRM model fit indices					
		a	b_1_	b_2_	b_3_	b_4_	M2 (df)	*p*	RMSEA	SRMRS	TLI	CFI
Unidimensional	Prev1	2.02	–2.78	–2.05	–1.25	–0.14	32.07 (2)	<0.01	0.05	0.02	0.98	0.99
	Prev2	1.92	–2.17	–0.97	–0.26	0.46						
	Prev3	1.72	–3.03	–2.04	–1.32	–0.69						
	Prev4	1.44	–3.39	–1.98	–0.97	–0.04						

*a = discrimination parameters; b = difficulty parameters.*

**FIGURE 2:**
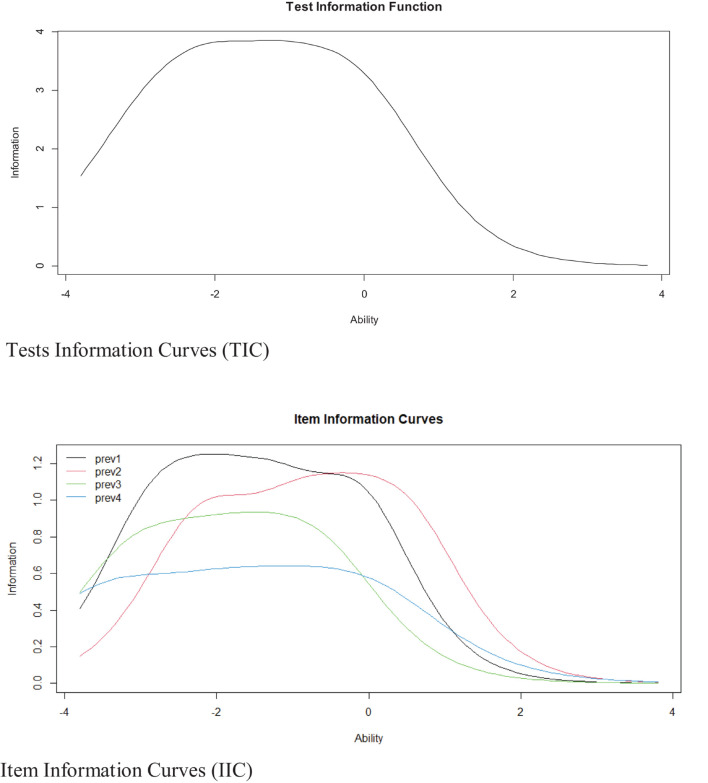
Item and test information curves for the scale.

Figure 2 shows the Information Curves for the five items and the scale as a whole (IIC and TIC respectively). The IIC shows that item 1 is the most accurate item of the scale for assessing the latent trait. In addition, the TIC shows that the test is most reliable (accurate) in the range of the scale between –3 and 1.

### Validity Based on the Relation With Other Variables

**FIGURE 3:**
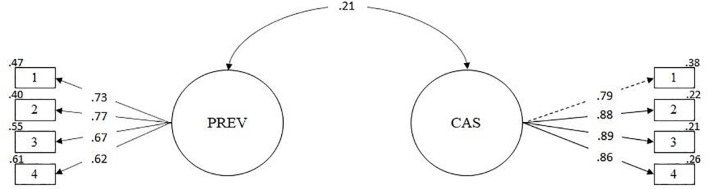
Modelo de relación con otros constructos.

Based on the literature review, an SEM model was proposed to evaluate the relationship between preventive behaviors and the level of COVID-19 anxiety. It was observed that the model presents adequate fit indices (χ^2^ = 210.61; df = 19; *p* = 0.000; RMSEA = 0.044 [CI90% 0.039 –0.050]; SRMR = 0.037; CFI = 0.99; TLI = 0.99). Furthermore, the measurement models are adequately represented by their items (see Figure 3). Figure 3 also shows that preventive behaviors are positively related to COVID-19 anxiety (ρ = 0.21; *p* < 0.01). Based on these results, it can be concluded that the scale presents evidence of validity in relation to other variables.

## Discussion

As the COVID-19 pandemic evolves, the practice of preventive behaviors becomes increasingly important to contain disease transmission (Gallegos et al., 2020; Mat Dawi et al., 2021). Thus, it is important to have a measure to know how often people perform preventive behaviors against COVID-19 that are recommended by public health authorities globally. Therefore, in this study we assessed the MI of the PCIBS in Latin American countries as a measure of health behaviors related to COVID-19 prevention based on WHO recommendations.

First, it was found that the unidimensional five-item model did not present an adequate fit in most countries. Therefore, searching for a better fitting model, it was identified that item 5 (“How often do you stay at home when you do not feel well?”) presents the lowest factor loadings in every country. Ventura-León’s (2020) recommendations support the removal of item 5. The factor loadings for item 5 ranged from 0.25 to 0.53; therefore, squaring these values would suggest that between 6.25 and 28.09% of the variance of the item in each country is explained by the factor (COVID-19 prevention behaviors). These percentages are relatively small and suggest the presence of other more important preventive behaviors. In addition, considering that the general population was assessed, where the majority reported not having had COVID-19, it is possible that the behavior of staying at home in the presence of minimal symptoms was not significant. While in other circumstances, not leaving home except when necessary was at adequate frequency levels (Ozdemir et al., 2020; Shahnazi et al., 2020), the high levels of poverty and inequality in Latin American countries (Burki, 2020; Pablos-Méndez et al., 2020), could lead people to risk leaving their homes to work, even though they do not feel well. This highlights the importance of designing public health policies to address pandemics that are tailored to the circumstances of each Latin American population (Meda-Lara et al., 2021).

A four-item model (without the presence of item 5) was tested and contrasted with the original model. The results of the CFA support a unidimensional structure of the four-item model, where all items load significantly on the latent factor; furthermore, the factor loadings are above 0.50 in all groups, which is considered good (Tabachnick and Fidell, 1996). An important fact to take into consideration is that some RMSEA values of model 2 were higher than those recommended in countries such as Cuba, Colombia, Chile and Argentina (Kline, 2015; Schumacker and Lomax, 2015). However, this was to be expected, as the RMSEA tends to perform poorly in factor models with few degrees of freedom, such as a model comprised of five indicators (or items), even when the model is correctly specified (Kenny et al., 2015; Taasoobshirazi and Wang, 2016). Despite this, models with large RMSEA values and small degrees of freedom should not be discarded without examining other information, such as the values of the other fit indices (Kenny et al., 2015).

The above results allow us to propose a new version of the PCIBS in Spanish, called the PCIBS-4, consisting of four items that measure the frequency of preventive behaviors against COVID-19. These behaviors include frequent hand washing; avoiding touching the eyes, nose or mouth; covering the mouth or nose when coughing or sneezing; and maintaining a social distance of at least 1 m. Although there are criticisms regarding the psychometric quality of short scales, such as the PCIBS (Smith et al., 2000; Credé et al., 2012), their presence can be valuable. It is now increasingly popular and widespread to develop and use short measures to assess a broad set of behaviors in clinical and non-clinical contexts (Kruyen et al., 2013). Using short measures would save time in assessment and reduce associated costs (Kemper et al., 2019); moreover, their use has been found to increase study participation rates (Edwards et al., 2004) and reduce fatigue, as well as other negative reactions that could lead to lower data quality (Credé et al., 2012). Similarly, reliability in all countries was greater than 0.70 (ranging from 0.71 to 0.88), either using the alpha or omega coefficient, which is within the expected range based on the original study (α = 0.82). Thus, it is suggested that the PCIBS-4 is a consistent measure of preventive behaviors against COVID-19 in English and Spanish.

Having defined that the PCIBS-4 model is the best fitting model, its MI was examined among the 12 Latin American countries using two different methods (MG-CFA and CFA-MIAL). The result based on the MG-CFA method revealed that the configural and metric invariance of the PCIBS-4 were maintained; however, the scalar invariance was not. In this regard, a partial scalar invariance test was performed by releasing the intercepts of items 1 and 4, referring to regular hand cleaning with alcohol-based hand sanitizer or soap and water and avoidance of touching eyes, nose and mouth (Dimitrov, 2010). Evidence of partial invariance suggests that people in the countries assessed rate only two items of the PCIBS-4 equivalently, where items 1 and 4 are not invariant. This would indicate that participants from different countries place different importance on items 1 and 4 when assessing preventive behaviors against COVID-19. This could be associated with differences in the adoption of preventive behaviors between younger and older people, something that has not been assessed in this study. In this sense, it has been reported that younger people have a higher frequency of preventive behaviors than older people; also, while younger people perceive a higher risk of infection, older people tend to perceive a higher risk of death from the disease (Kim and Crimmins, 2020). Thus, it is important that future studies assess the impact of age on the adoption of preventive behaviors in different countries, with the additional aim of proposing appropriate intervention strategies for different age groups. The non-invariance of the intercepts of items 1 and 4 would also suggest that the observed differences in means between countries would not truly express differences at the latent level (Schnettler et al., 2017). However, as others point out, the presence of partial invariance might be sufficient to compare means between different groups (Whisman and Judd, 2016), as in practice it is difficult for full MI to be present (Schnettler et al., 2017). However, because the PCIBS-4 is constituted by four items, it was considered risky to suggest not considering items 1 and 4 for the comparison of preventive behaviors against the COVID-19 between countries, given that the scale would be constituted by only two items. In this sense, it was considered worth noting that the PCIBS-4 reaches metric invariance, which allows for comparing the relationship of each item with the construct measured between the groups, but does not allow for comparing the total score.

Considering that there is insufficient evidence to support scalar invariance using the MG-CFA method, it was feasible to test the CFA-MIAL method (Marsh et al., 2018). While this method does not allow for testing partial invariance, it does provide information about the approximate MI and the range of factor means across a large number of groups; moreover, this method was developed specifically to test the comparability of factor means across a large number of groups (Jang et al., 2017). Particularly, this method is beneficial when comparing groups of countries, where the presence of non-invariance is expected due to cultural differences between countries (Muthén and Asparouhov, 2018). Even still, recent studies have shown that the CFA-MIAL method is feasible for testing MI among more than 90 groups (Munck et al., 2018). For the CFA-MIAL method to work properly, two requirements must be met: an acceptable fit of the configurational model based on the MG-CFA method and obtaining a pattern of invariance in the data, with only a minority of the parameters demonstrating non-invariance. In general, both requirements were achieved by applying a one-factor model for the PCIBS-4. In this regard, the CFA-MIAL indicated that the factor structure of the PCIBS-4 is invariant for factor loadings and intercepts. Despite the advantages of the CFA-MIAL, some authors suggest that this method is relatively new, so, the decision criterion, referring to less than 25% of the parameter estimates being non-invariant, should be considered with caution until more evidence can be derived from simulation studies verifying this cut-off point (Jang et al., 2017). Still, the findings based on the CFA-MIAL allow us to meaningfully compare factor means across countries based on the assumption of approximate MI.

Based on the above, the means of the PCIBS-4 were compared among the 12 Latin American countries. It was observed that, in general, the differences were irrelevant; however, Mexico and Peru presented the highest frequency of preventive behaviors against COVID-19 compared to the rest of the countries. This is not surprising, as both countries have been among the most affected throughout the pandemic in Latin America (Garcia et al., 2020). In contexts where the pandemic has had a more negative impact, as in the aforementioned countries, a higher perceived risk of infection is observed, which in turn is likely associated with a greater adoption of prevention behaviors against the disease (Bowman et al., 2021). A previous study in Mexico also reported similar results, indicating that the general population of that country performed an average of 13.5 preventive actions against COVID-19 from a range of 0 to 19 (Sánchez-Arenas et al., 2021). In the Peruvian case, although there are no data on the frequency of preventive behaviors against COVID-19, a study with health professionals indicated that 31.5% presented high levels of preventive practices (Rivera-Lozada et al., 2021). On the other hand, Uruguay was one of the countries with the lowest frequency of preventive behaviors. The Uruguayan government’s measures have allowed it to adequately manage the pandemic, making it one of the Latin American countries with the lowest rate of diagnosed cases and deaths from COVID-19 (Taylor, 2020), which, in turn, was previously associated with a lower levels of fear of COVID-19 as reported in a cross-cultural study (Caycho-Rodríguez et al., 2021e). All of this could be related to the lower presence of preventive behaviors. Likewise, adherence to preventive behaviors against COVID-19 influences the incidence of diagnosed cases and deaths from COVID-19 (Sánchez-Arenas et al., 2021). Efforts to engage in preventive behaviors have been important. For example, in Mexico, the number of diagnosed cases and observed deaths are lower than estimated in the worst-case scenario of the pandemic based on statistical models (Ramírez-Valverde and Ramírez-Valverde, 2021). Similar results have been shown for the Peruvian case (Córdova and Santa María, 2021).

Another contribution of this study is that it provided, for the first time, information on the properties of the items of the PCIBS-4 in Spanish based on IRT in a large sample of people living in Latin American countries. In this sense, the four items present increasing difficulties, indicating that a person with low frequency of preventive behaviors related to COVID-19 will tend to choose the lower response alternatives; while people with a higher frequency will choose higher response alternatives. This is expected and appropriate for instruments of this type, since it would reflect the fact that the content of the items allows for the response of all the alternatives without losing information. Likewise, all items have high values in the discrimination parameters. This suggests that the PCIBS-4 will be able to easily differentiate between the responses of a person with a higher frequency of preventive behaviors and one with a moderate or low frequency. The results also indicate that the PCIBS-4 could better and more accurately assess the frequency of preventive behaviors against COVID-19 in people with low and very low levels of the variable, where items 1 and 2 are the ones which best take advantage of this characteristic. In this sense, it is more likely that a person with a high and very high frequency of preventive behaviors would show similar scores and would mark mainly the last response alternatives in all items. Therefore, the scale would provide little information about these people, as it would be more appropriate to detect people who present a very poor practice of preventive behaviors. If we wanted to improve the scale’s ability to discriminate between people with average or higher levels of prevention, we could add items describing more ‘comprehensive’ prevention behaviors.

Regarding the evidence of validity in relation to other variables, it was found that COVID-19 anxiety was positively related to preventive behaviors against COVID-19, although the relationship is low. This finding is in accordance with the literature on the subject (Alrubaiee et al., 2020; Kwok et al., 2020; Velikonja et al., 2020; Wong et al., 2020). In this regard, greater preventive behavior is observed in those people who experience greater anxiety about COVID-19. A possible explanation is that more anxious people present greater mental distress and higher perceived vulnerability to infectious diseases, leading to a higher frequency of preventive behaviors (Taylor, 2019). This has also been observed during the H1N1 influenza pandemic in 2009, where people who saw themselves at high risk of infection were more likely to wash their hands and get vaccinated (Gilles et al., 2011; Taha et al., 2014). The increase in the number of cases of COVID-19 and increased concern for personal safety has caused anxiety to become an important psychological factor influencing how a person responds to a viral outbreak, such as COVID-19 (Taylor, 2019). Still, the low correlation between the variables makes further studies necessary to understand how anxiety about COVID-19 is specifically associated with preventive behaviors in the face of COVID-19 (Asmundson and Taylor, 2020).

One of the main strengths of the study is the use of a large sample size from different countries. This allowed for a greater variability among the participants and to extend the generalization of the findings to a multicultural context, which is Latin America. However, there are also some limitations that should be considered when interpreting the results. First, although the study included samples from twelve Latin American countries (mostly from South America), future research should examine whether the findings generalize to other countries in the Americas and the world. Second, participants were selected through non-probability convenience and snowball sampling in all countries. This may limit the generalizability of the results to the general population of different regions within the same country. Third, the number of participants differed between countries, which may have resulted in not obtaining the same levels of variability in the way COVID-19 preventive behaviors are expressed. Fourth, due to the online nature of the survey, the study was basically aimed at people with internet access, generally residing in urban areas in each of the countries. This may further limit the generalizability of the results. Fifth, the study did not examine possible differences in the frequency of preventive behaviors between subgroups within each country (for example, between males and females or between those who did and did not have COVID-19). Sixth, in each country, the majority of participants had graduated from university. In this regard, future studies could evaluate the MI of the PCIBS-4 in matched populations with respect to educational level. The university-educated population does not necessarily represent the majority and tends to have more privileges (such as greater purchasing power and access to better services). This may influence the degree to which individuals exhibit COVID-19 preventive behaviors compared to others. Seventh, because participants in every country were 18–80 years old, the usefulness of the PCIBS-4 in younger samples (including children and adolescents) is unclear. Eighth, due to the self-report nature of the PCIBS-4, the data may be vulnerable to social desirability bias. In this regard, participants were relied upon to reflect on and report the extent to which they engage in each of the behaviors assessed. It should be considered that self-reported behaviors do not always correspond to actual behaviors, as participants may have difficulty or do not want to report accurate estimates (Prince et al., 2008). Thus, future studies should integrate assessments of participants’ actual prevention behavior. Despite the presence of these limitations, the findings are encouraging with respect to the MI of the PCIBS-4 in Spanish in different countries.

The findings provide evidence that the PCIBS-4 is a unidimensional self-report measure that is reliable and invariant across the twelve participating Latin American countries. In addition, the PCIBS-4 presents evidence of validity based on the relationship with other variables, such as COVID-19 anxiety. In the current pandemic context, there is a need to understand different behavioral patterns in a variety of cultural contexts. In this sense, assessing the psychometric properties and cross-cultural utility of the different measures available contributes to a better understanding of the theoretical underpinnings of these instruments and their usefulness. At a theoretical level, having a unidimensional measure allows for the assessment of a specific construct, such as preventive behaviors against COVID-19 infection, and not other behaviors related to other types of conditions during the pandemic, such as smoking (Chen, 2020). In this sense, if any of the PCIBS-4 items assess not only preventive behaviors against COVID-19 infection, but also other behaviors, then the total score should also include information on the latter and, therefore, the interpretation of the PCIBS-4 would be wrong (Ziegler and Hagemann, 2015). In addition, more information has been provided on the role of COVID-19 anxiety in prevention behaviors and measures to take in the face of the disease, contextualized to Latin American countries and complementing what has been reported in other countries. On a practical level, it is hoped that the findings will be of interest to social and health scientists, as well as those professionals directly involved in public health decision making. Thus, the PCIBS-4 appears to be sufficiently useful for public health policy makers as an instrument to monitor compliance with preventive behaviors in the participating Latin American countries. In this sense, it was concluded that there are irrelevant differences between countries with respect to the frequency of preventive behaviors against COVID-19 in the 12 countries included in the study, where Mexico and Peru presented the highest frequency of preventive behaviors against COVID-19 compared to the rest of the countries. Even so, it seems that the negative impact of COVID-19 in Latin America, expressed in the number of diagnosed cases and deaths, has led people in the countries evaluated to have a high frequency of preventive behaviors. Thus, it appears that the incidence of COVID-19 cases and deaths has impacted adherence to COVID-19 preventive behaviors, along with variability in risk perception within and between countries (Clark et al., 2020). Thus, knowing the frequency with which individuals perform preventive behaviors against COVID-19, via the PCIBS-4, is important to carry out intervention actions and adapt prevention guidelines in each of the countries, based on identifying the groups of people who least frequently perform preventive behaviors. Thus, the PCIBS-4 can be a tool to support pandemic control strategies at the regional level. Researchers can use the PCIBS-4 to examine the frequency of COVID-19 preventive behaviors among different cultural groups. Also, having a cross-culturally validated measure gives greater confidence to interpret mean differences detected with the PCIBS-4 as true and not the product of measurement error. It could also provide a measure of the effectiveness of interventions to promote preventive behaviors. Building confidence in the effectiveness of these behaviors could lead to more frequent participation and reduce the need for intrusive government interventions. Good public health planning and policy development could provide incentives for people to practice the preventive behaviors that can help mitigate the impact of the pandemic.

## Data Availability Statement

The raw data supporting the conclusions of this article will be made available by the authors, without undue reservation.

## Ethics Statement

The studies involving human participants were reviewed and approved by Universidad Privada del Norte (registry number: 20213002). The patients/participants provided their written informed consent to participate in this study.

## Author Contributions

TC-R, LV, PV, and CC-L provided initial conception, organization, and main writing of the text. LV and PV analyzed the data and prepared the all figures and tables. AV-V, DS-A, MR-B, MW, CR-J, RP-C, MG, MC, PM, DP, RM-H, AS-P, ML-R, IF, CF-M, AF, DP-C, IC-R, RC, BT, and WG were involved in data collection for their respective countries and acted as consultants and contributors to research design, data analysis, and text writing, read and approved the draft. All the authors contributed to the article and approved the submitted version.

## Conflict of Interest

The authors declare that the research was conducted in the absence of any commercial or financial relationships that could be construed as a potential conflict of interest.

## Publisher’s Note

All claims expressed in this article are solely those of the authors and do not necessarily represent those of their affiliated organizations, or those of the publisher, the editors and the reviewers. Any product that may be evaluated in this article, or claim that may be made by its manufacturer, is not guaranteed or endorsed by the publisher.
